# Processed Multiparameter Electroencephalogram-Guided General Anesthesia Management Can Reduce Postoperative Delirium Following Carotid Endarterectomy: A Randomized Clinical Trial

**DOI:** 10.3389/fneur.2021.666814

**Published:** 2021-07-12

**Authors:** Na Xu, Li-Xia Li, Tian-Long Wang, Li-Qun Jiao, Yang Hua, Dong-Xu Yao, Jie Wu, Yan-Hui Ma, Tian Tian, Xue-Li Sun

**Affiliations:** ^1^Department of Anesthesiology, Xuanwu Hospital, Capital Medical University, Beijing, China; ^2^Department of Neurosurgery, Xuanwu Hospital, Capital Medical University, Beijing, China; ^3^Department of Vascular Ultrasound, Xuanwu Hospital, Capital Medical University, Beijing, China

**Keywords:** delirium, carotid endarterectomy, electroencephalogram-guided, general anesthesia, cerebral perfusion, monitoring

## Abstract

**Background:** Patients undergoing carotid endarterectomy (CEA) for severe carotid stenosis are vulnerable to postoperative delirium, a complication frequently associated with poor outcome. This study investigated the impact of processed electroencephalogram (EEG)-guided anesthesia management on the incidence of postoperative delirium in patients undergoing CEA.

**Methods:** This single-center, prospective, randomized clinical trial on 255 patients receiving CEA under general anesthesia compared the outcomes of patient state index (PSI) monitoring [SEDLine Brain Function Monitor (Masimo, Inc, Irvine, CA)] (standard group, *n* = 128) with PSI combined with density spectral array(DSA) -guided monitoring (intervention group, *n* = 127) to reduce the risk of intraoperative EEG burst suppression. All patients were monitored by continuous transcranial Doppler ultrasound (TCD) and near-infrared spectroscopy (NIRS) to avoid perioperative cerebral hypoperfusion or hyperperfusion. According to the surgical process, EEG suppression time was calculated separately for three stages: S_1_ (from anesthesia induction to carotid artery clamping), S_2_ (from clamping to declamping), and S_3_ (from declamping to the end of surgery). The primary outcome was incidence of postoperative delirium according to the Confusion Assessment Method algorithm during the first 3 days post-surgery, and secondary outcomes were other neurologic complications and length of hospital stay.

**Results:** There were no episodes of cerebral hypoperfusion or hyperperfusion according to TCD and NIRS monitoring in either group during surgery. The incidence of postoperative delirium within 3 days post-surgery was significantly lower in the intervention group than the standard group (7.87 *vs*. 28.91%, *P* < 0.01). In the intervention group, the total EEG suppression time and the EEG suppression time during S2 and S3 were shorter (Total, 0 “0” *vs*. 0 “1.17” min, *P* = 0.04; S_2_, 0 “0” *vs*. 0 “0.1” min, *P* < 0.01; S_3_, 0 “0” *vs*. 0 “0” min, *P* = 0.02). There were no group differences in incidence of neurologic complications and length of postoperative hospital stay.

**Conclusion:** Processed electroencephalogram-guided general anesthesia management, consisting of PSI combined with DSA monitoring, can significantly reduce the risk of postoperative delirium in patients undergoing CEA. Patients, especially those exhibiting hemodynamic fluctuations or receiving surgical procedures that disrupt cerebral perfusion, may benefit from the monitoring of multiple EEG parameters during surgery.

**Clinical Trial Registration:** www.ClinicalTrials.gov, identifier: NCT03622515.

## Key Points

- **Question:** Can EEG-guided general anesthesia management reduce the risk of postoperative delirium in high-risk patients undergoing CEA?
- **Findings:** Processed electroencephalography-guided anesthesia management, including PSI combined with DSA monitoring, can significantly reduce postoperative delirium incidence in patients undergoing CEA.
- **Meaning:** This randomized clinical trial suggests that slight changes in anesthetic management may play a major role in modulating postoperative delirium risk in patients undergoing CEA under general anesthesia. Vulnerable patients, especially those exhibiting hemodynamic fluctuations or undergoing surgical procedures that disrupt cerebral perfusion, may benefit from the monitoring of multiple EEG parameters during surgery.

## Introduction

Carotid endarterectomy (CEA) is the gold standard treatment for patients with severe carotid stenosis (CS) to reduce the risk of stroke (1–4). The safety and long-term efficacy of CEA for the management of carotid artery disease has been demonstrated in large randomized controlled trials (5–8). However, cerebral blood supply may be severely disrupted by anesthesia and surgical manipulations during CEA, and cerebral function is highly vulnerable to even brief changes in oxygen and blood supply. Cerebral vascular diseases may further increase the risk of perioperative neurological dysfunction. For instance, there is a significant association between carotid artery stenosis and postoperative delirium (9, 10), an acute state of mental confusion defined by alterations in attention, consciousness, and disorganized thinking (11, 12). Postoperative delirium (POD) is a common yet serious geriatric syndrome that afflicts 10–60% of patients after major surgery (13–16) and up to 91% of the critically ill (17). Further, POD is associated with worse early and long-term prognosis as well as greater healthcare costs (11, 14, 17–19). Therefore, the United Kingdom National Institute for Health and Care Excellence, the American Geriatric Society, the American College of Surgeons, and the American Society of Anesthesiologists have all identified the prevention of postoperative delirium as a public health priority (20–23).

Risk factors for delirium are usually divided into predisposing and precipitating (24). Predisposing factors include preoperative vulnerabilities, while precipitating factors are potentially reversible events occurring throughout the perioperative period. POD incidence increases with longer cumulative intraoperative electroencephalogram (EEG) suppression duration (25, 26), and processed EEG monitoring during surgery may lower POD rate and prevent or minimize EEG suppression by using minimal anesthetic doses (27–31). A burst suppression pattern on the EEG indicates severe inhibition of neuronal activity and metabolic rate. Patients with severe CS are prone to vascular cerebral injury, and may therefore be particularly vulnerable to EEG suppression (32, 33). It is not clear, however, whether processed EEG-guided anesthesia management can reduce POD incidence in patients undergoing CEA and if this effect depends on decreasing EEG suppression.

The objectives of this study were to investigate the effect of processed EEG-guided anesthesia management on the incidence of POD in patients receiving CEA under general anesthesia. The primary hypothesis was that processed EEG-guided anesthesia management can effectively reduce the likelihood of the incidence of POD during the first 3 days following CEA.

## Methods

### Research Design

This is a single-center, prospective, randomized clinical trial with two parallel arms. The research protocol was approved by the Ethics Committee of Xuanwu Hospital of Capital Medical University (LYS[2018]053) and written informed consent was obtained from all subjects participating in the trial. The trial was registered prior to patient enrollment at clinicaltrials.gov (NCT03622515, Principal investigator: Na Xu, Date of registration: August 8, 2018). All surgeries were conducted at Xuanwu Hospital (Beijing, China). Results are reported according to CONSORT guidelines.

### Participants

Study candidates were screened by investigators the day before surgery (or on Friday for those scheduled for surgery the following Monday). We recruited patients at our institution from August 2018 to December 2019. All surgeries were performed by the same surgical and anesthesia team. Inclusion criteria included patients scheduled to undergo elective CEA under general anesthesia, fluency in Chinese Mandarin, an anticipated length of hospital stay over 3 days after surgery, and willingness to comply with the research protocol. Exclusion criteria were as follows: (1) declining preoperative condition (such as unstable angina, acute myocardial infarction, and heart function NYHA III to IV) within 4–6 weeks before surgery, (2) severe hepatic dysfunction (Child–Pugh grade C) or renal failure (requirement for renal replacement therapy), (3) history of schizophrenia, Parkinson's disease, or traumatic brain injury, (4) inability to perform neurocognitive testing, (5) preoperative delirium according to the Confusion Assessment Method (CAM) algorithm (34), (6) preoperative cognitive impairment according to the Chinese Mini-Mental State Examination (MMSE) corrected for education level (illiterate ≤ 19 points, 1–6 years of primary school ≤ 22 points, middle school or above ≤ 26 points) and Montreal Cognitive Assessment (MoCA) score (illiterate ≤ 13 points, primary school ≤ 19 points, middle school or above ≤ 24 points) (35, 36), (7) preoperative depression according the Self-rating Depression Scale (SDS, score > 41 points) or preoperative anxiety according to the Self-rating Anxiety Scale (SAS, score > 41 points) (37, 38), (8) change in surgical procedure after anesthesia, (9) return to the intensive care unit following surgery, (10) conditions that caused severe hemodynamic fluctuations (such as severe allergic reactions or major bleeding), and (11) accidental discharge.

### Baseline Data Collection

Baseline data included demographics, co-morbidities, and relevant physical and laboratory findings. Preoperative physical condition was evaluated using the American Society of Anesthesiologists (ASA) Physical Status Classification System (39). Activities of daily living were assessed using the Barthel Index (score range 0–100, with higher score indicating better independent function) (40). Cognitive functions were assessed using the MMSE and MoCA. Anxiety and depression were assessed using the SAS and SDS because several studies have identified preoperative depression (41–43) and anxiety (44) as risk factors for postoperative delirium incidence or duration. Delirium status was assessed with the CAM. Pre-surgical tests of baseline general cognition, delirium, anxiety, and depression were conducted by a neuropsychologist blinded to group allocation (see below).

### Randomization and Blinding

Patients were assigned to intervention and control arms before surgery using a computerized random number generator at a 1:1 ratio. A seed was not specified, and blocks were not used in randomization. Randomization was conducted after patient consent for research participation during the preoperative interview. Both patients and research associates conducting preoperative testing and postoperative outcome assessments were also blinded to group assignment.

### Anesthesia and Surgery

General anesthesia was induced in all patients by intravenous etomidate 0.15 mg·kg^−1^, and sufentanil 0.2 mg·kg^−1^, and maintained by continuous propofol infusion. In the standard monitoring group, propofol dosage was 50–80 mcg·kg^−1^·min^−1^, while in the intervention group, dosage was adjusted according to sedative depth as described in the next section (**Intervention**). All patients received remifentanil and dexmedetomidine as well as rocuronium (0.6 mg·kg^−1^) or cisatracurium (0.15 mg·kg^−1^) injection for muscle relaxation. Patients were mechanically ventilated with FiO_2_ 50%, and SaO_2_ was maintained at more than 95%. Goal-directed fluid and vasoconstrictive drug therapies were conducted to maintain stroke volume variability below 13% and regulated patients' blood pressure within 180 mmHg according to transcranial Doppler ultrasound (TCD, China Shenzhen Delica Medical Equipment, ShenZhen, China) and near-infrared spectroscopy (NIRS, Cas Medical Systems, Inc, Branford, Connecticut, USA). Intraoperative warming devices were used to maintain nasopharyngeal temperature between 36°C and 37°C. Perioperative care was standardized according to institutional routines for all patients.

All CEA procedures were performed by the same team of 4 experienced neurosurgeons who have worked for more than 15 years. As there is a correlation between intraoperative hypotension and incidence of delirium (45, 46), all patients were monitored by TCD and NIRS during CEA to minimize the risks of perioperative cerebral hypoperfusion and hyperperfusion. Regional cerebral oxygenation (rSO_2_) was also monitored by NIRS and mean flow velocity of the middle cerebral artery (MFV_MCA_) by TCD. The NIRS probes were placed on the bilateral forehead. Cerebral ischemia was deemed to have occurred if the ipsilateral MFV_MCA_ was reduced by >50% compared to baseline or rSO_2_ decreased by 20% from baseline during the clamping process (47–50). An increase in MFV_MCA_ of 100% after carotid declamping compared to baseline was considered indicative of cerebral hyperperfusion (51). Anesthesiologists managed cerebral hypoperfusion or hyperperfusion during CEA by regulating the patient's blood pressure, which was elevated or reduced by 10% before and after declamping the carotid artery, respectively. Carotid shunting was conducted based on TCD monitoring and the surgeon's discretion.

### Intervention

During surgery, patients from both the groups were monitored using the SEDLine Brain Function Monitor (Masimo, Inc, Irvine CA), which uses symmetrical bifrontal electrodes to measure four channels of raw EEG data with separate displays for electromyogram (EMG), artifacts (e.g., patient motion), burst suppression ratio (BSR), and density spectral array (DSA). The SEDLine monitor also estimates sedative depth from digital EEG waves using a proprietary algorithm and displays it as a dimensionless parameter called the patient state index (PSI, ranging 0–100, with 100 indicating wakefulness and 0 isoelectric EEG) (52). Even though there are some raw EEG patterns clearly visible on the spectrogram plots, we are nowhere close to being able to describe the cerebrum depending on the information obtained from the processed EEG. Therefore, in the intervention group, we visually analyzed and inspected DSA as an intervention indicator for EEG burst suppression rather than EEG suppression waveforms (53, 54). Whenever the DSA indicated burst suppression, propofol dosage was reduced 5 mcg·kg^−1^·min^−1^, as clinically permitted. In order to prevent intraoperative awareness, if lowering propofol was found to cause PSI to reach 60, but DSA still showed burst suppression, we stopped adjusting propofol and adjusted blood pressure according to the stage of surgery to ensure cerebral perfusion. For instance, blood pressure was elevated by 5% before declamping the carotid artery or decreased by 5% after declamping. In the standard group, the monitor screen was masked, all EEG data and spectrograms were blinded, and only PSI values were displayed. Anesthesiologists performed anesthesia by conventional methods and maintained the PSI at 25–50 in the standard group.

### Outcome Assessments

#### Measurement of Delirium

Delirium was assessed by trained research team members preoperatively and daily on the first three postoperative days using the Chinese version of CAM algorithm, which has demonstrated good reliability and validity among the Chinese population (55). Postoperative visits were conducted between 10:00 A.M. and 16:00 P.M. at the patient's bedside. Delirium was defined by acute onset with fluctuating course, inattention, disorganized thinking, and (or) altered level of consciousness.

#### Measurement of EEG Suppression

The raw EEG data, PSI, and BSR were obtained from all patients using the SEDLine monitor. The EEG recordings were initiated from anesthesia induction, and ended at the completion of surgical manipulation. All EEG data were then edited and saved. The experienced neurophysiologists independently reviewed the intraoperative EEG traces acquired by the SEDLine® monitoring system for the entire duration of the operation, recorded whether or not burst suppression was present, and calculated the cumulative duration of EEG suppression in minutes. The neurologists recorded an epoch as having a burst suppression pattern if there was at least 5 s of suppression of the EEG tracing present in a given 30 s epoch (56). We recorded the cumulative duration of total EEG suppression and the duration of EEG suppression for three surgical stages: S_1_ (from anesthesia induction to carotid artery clamping), S_2_ (from clamping to declamping), and S_3_ (from declamping to the end of surgery).

The anesthesiologists received training by neurophysiologists in reading and interpreting the DSA before the study was initiated to ensure that all anesthesiologists could reach 100% consensus on the interpretation of the DSA. Additionally, the training process was repeated at 6-month intervals during the study.

#### Magnetic Resonance Image Data Acquisition

Cerebral microembolization is a significant contributor to postoperative delirium (57, 58). Therefore, magnetic resonance imaging examinations were conducted before and 24 h following surgery using a Clinical 3-Tesla whole-body MR imager (Verio; Siemens Medical Solutions, Erlangen, Germany). In accordance with previous studies (59–62), new ischemic cerebral lesions were defined as hyperintense regions on post-intervention diffusion-weighted images that were not present on pretreatment images. Ischemic lesions (number and total volume of hyperintense regions) were evaluated by a radiologist and neurologist both blinded to the research protocol, and disagreements were resolved by consensus.

#### Measurement of Anesthetic Doses

To determine whether processed EEG-guidance resulted in propofol dosage reduction, we calculated the cumulative doses of all anesthetics according to intraoperative electronic medical records.

#### Measurement of Vital Signs

Mean arterial blood pressure (MAP), PSI, end-expiratory carbon dioxide partial pressure (P_ET_CO_2_), bilateral rSO_2_, and bilateral MFV_MCA_ were recorded at three time-points, immediately after general anesthesia induction and before carotid artery clamping as baseline (T_1_), immediately after clamping (T_2_), and after declamping and subsequent stabilization of cerebral perfusion (T_3_).

#### Measurement of Secondary Outcomes

Physical and neurological examinations were conducted by a neurologist blinded to group allocation before and for the first 3 days after surgery. Examinations included evaluation of neurological deficits according to the National Institutes of Health Stroke Scale. From the beginning of anesthesia to 3 days after surgery, we also monitored adverse events such as intraoperative movement or awareness. Finally, length of hospital stay was recorded.

### Statistical Analysis

#### Postoperative Outcome Analysis

The Kolmogorov–Smirnov test was used to check for the normality of all continuous variables. Continuous datasets with a normal distribution were compared by independent-samples *t*-test, and continuous datasets with non-normal distributions by independent-samples Mann–Whitney *U*-tests. Categorical data were compared by χ^2^ test or Fisher exact test as indicated. Measurement data at each time point were compared between the two groups by analysis of variance with repeated measures (RT-ANOVA). Normally distributed continuous variables are reported as mean ± standard deviation (s.d.) and non-normally distributed continuous variables as median (interquartile range, i.q.r.).

Statistical analyses were conducted using SPSS® Version 21(IBM Inc. Chicago, IL, USA). All tests were two-tailed, and a two-sided *P* < 0.050 was considered statistically significant.

#### Sample Size Calculation

We calculated the sample size based on the expected incidence of the primary outcome (postoperative delirium). In a pilot study, 20 patients meeting the inclusion criteria were included and randomly divided into an intervention group and standard care group, with 10 patients in each group. One patient in the standard care group had postoperative delirium, whereas patients in the intervention group did not have any postoperative neurological complications. Therefore, postoperative neurological complications rates where 0% in the interventional group (to avoid errors, the incidence was increased of 0.5% for calculating the sample size) and 10% in the standard group. We set type I error α = 0.05 and the type II error β = 0.1. From this, the sample size of each group was estimated at 91 cases using the sample size software PASS11 (NCSS, Caseville, Utah, United States). Assuming a 20% loss to follow-up, we had planned to enroll at least 220 patients undergoing CEA.

## Results

### Patient Recruitment

**Figure 1:**
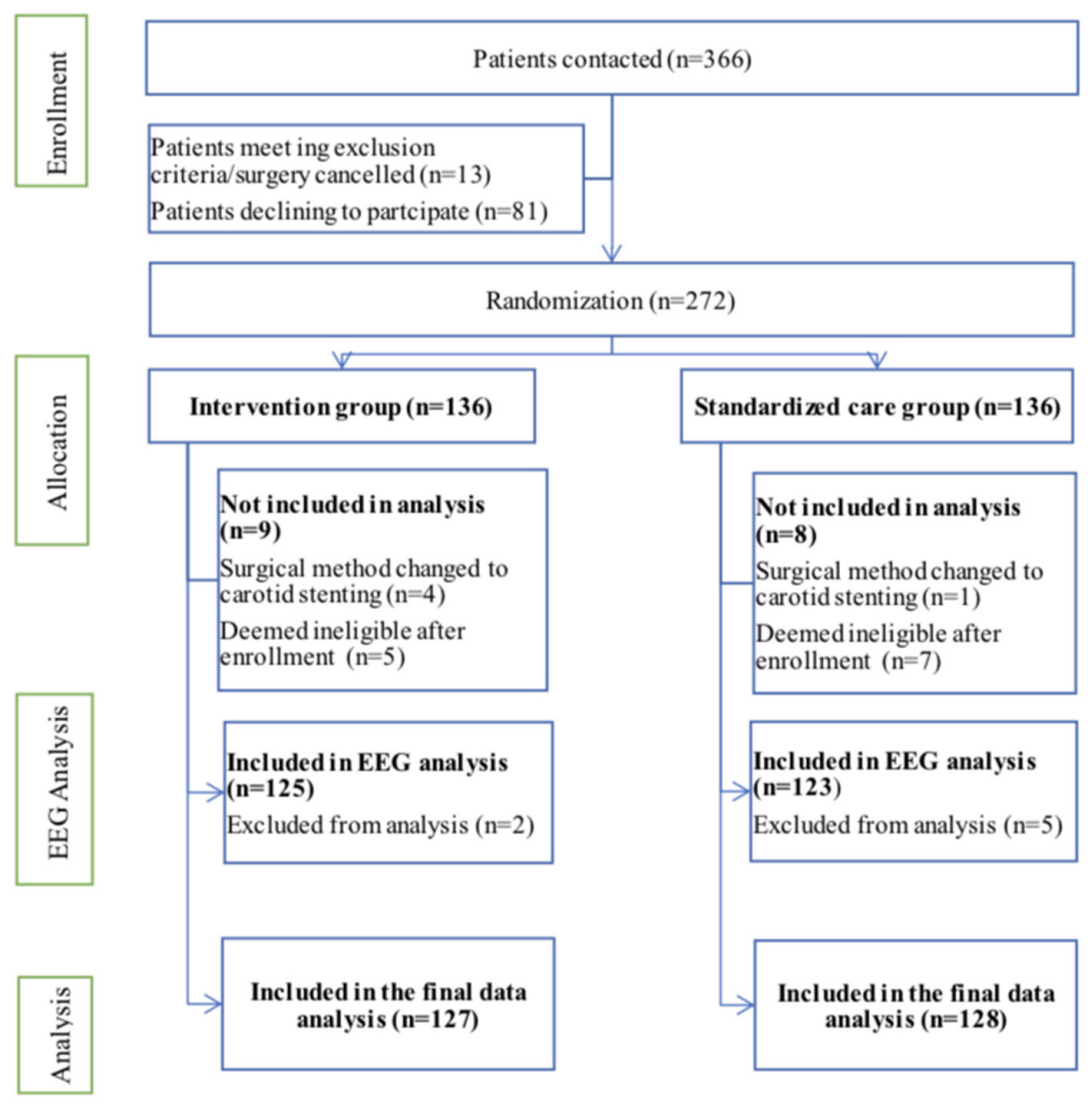
Flow diagram depicting patient recruitment for this clinical trial. EEG, electroencephalography.

This clinical trial was conducted from August 2018 to December 2019. Overall, 366 patients were deemed eligible and 272 patients were included, with patients randomly and equally divided into processed EEG-guided anesthetic management (intervention) and standard anesthesia care groups (Figure 1). Anesthesiologists adhered to the protocol for all 136 patients assigned to the intervention group. During the study period, five surgeries were changed to carotid stenting (four cases in the intervention group and one in the standard care group, and they did not receive any of the study intervention in either group), 10 cases did not complete the postoperative CAM test (five cases in each group), and two cases in the standard care group were transferred to the intensive care unit after surgery. Therefore, 255 patients were included in the final analysis, 127 in the intervention group and 128 in the standard care group (Figure 1). Preoperative demographic, surgical, and intraoperative variables for each group are summarized in Tables 1, 2. Baseline variables, such as age, sex ratio, preoperative co-morbidity, and cognitive and mood assessments, among others (Table 1), and intraoperative variables, such as surgery duration, anesthesia duration, and clamping duration, among others, were well-matched between the groups (all *P* > 0.05) (Table 2). In addition, intraoperative anesthesia dose did not differ between groups.

**Table 1:** Patient demographic and clinical information.

**Variable**	**Intervention group (*n* = 127)**	**Standard group (*n* = 128)**	***P*-value**
Age (y)^††^	62.31 ± 8.02	63.16 ± 7.17	0.37
Sex ratio (F:M)^§§^	18:109	15:113	0.56
Height (cm)^††^	168.74 ± 6.98	169.55 ± 6.31	0.33
Body weight (kg)^††^	71.61 ± 10.03	71.88 ± 10.16	0.83
BMI (kg/m^2^)^††^	25.11 ± 2.80	24.95 ± 2.81	0.65
**Preoperative co-morbidity**			
Hypertension^##^	92 (72.44)	86 (67.19)	0.36
Diabetes^##^	42 (33.07)	42 (32.81)	0.96
Coronary artery disease^##^	27 (21.26)	28 (21.88)	0.90
Previous stroke^##^	72 (56.69)	69 (53.91)	0.66
ASA fitness grade (III: IV)^§§^	37:90	31:97	0.38
**Preoperative level of function and mood**			
Barthel Index score*^††^	98.94 ± 3.79	99.35 ± 3.18	0.45
Education (y)^††^	9.93 ± 3.96	9.44 ±4.41	0.35
MMSE score^†††^	27.41 ± 2.06	27.09 ± 2.54	0.26
MoCA score^†††^	23.98 ± 2.53	23.63 ± 3.31	0.34
SDS score^‡^^††^	23.65 ± 4.99	24.45 ± 5.60	0.23
SAS score^‡^^††^	23.56 ±4.37	23.80 ± 4.92	0.67

Values in parentheses are percentages unless indicated otherwise; values are expressed as mean ± standard deviation (s.d.) or median (interquartile range, i.q.r.). *Score ranges from 0 to 100, with higher score indicating better function.

† *Score ranges from 0 to 30, with higher score indicating better function.*

‡ *Score ranges from 20 to 80, with higher score indicating worse mood. BMI, body mass index; MMSE, Mini-Mental State Examination; MoCA, Montreal Cognitive Assessment; SDS, Self-rating Depression Scale; SAS, Self-rating Anxiety Scale.*

§§ *The P-value is from the χ^2^ test, except.*

†† *The P-value is from the independent-samples t-test.*

## *The P-value is from the independent-samples Mann–Whitney U-test*.

**Table 2:** Intraoperative variables.

**Variable**	**Intervention group (*n* = 127)**	**Standard care group (*n* = 128)**	***P*-values**
Surgery duration (min)^*^^##^	135 (73.5)	139.5 (100.25)	0.50
Anesthesia duration (min)^*^^##^	213 (71.5)	216 (98.75)	0.75
Clamping duration(min)^*^^##^	38 (24.75)	35.5 (32)	0.94
Type of surgery (CEA: CEA+CAS)^§§^	91:36	87:41	0.52
Total fluid infusion(ml)^*^^##^	1,100 (400)	1,100 (500)	0.73
Crystal liquid (ml)^*^^##^	900 (400)	1,000 (400)	0.66
Urine output(ml)^*^^##^	700 (600)	700 (600)	0.86
Estimated blood loss(ml)^*^^##^	20 (20)	20 (20)	0.24
**Intraoperative drugs**			
Propofol (mcg/kg/min)^*^^##^	52.13 (14.11)	52.45 (15.98)	0.58
Remifentanil (mcg/kg/min)^*^^##^	0.20 (0.08)	0.21 (0.06)	0.32
Dexmedetomidine (mcg/kg/h)^*^^##^	0.29 (0.12)	0.27 (0.11)	0.48

*Values in parentheses are percentages unless indicated otherwise; values are mean ± s.d. and*

* *median (i.q.r.). CEA, carotid endarterectomy; CAS, carotid artery stenting.*

§§ *The P-value is from the χ^2^ test, except.*

## *The P-value is from the independent-samples Mann–Whitney U-test*.

### Incident Delirium

The incidence of postoperative delirium was significantly lower among patients receiving processed EEG-guided anesthesia management compared with that in the standard care group [10 of 127 (7.87%) *vs*. 37 of 128 (28.91%), *P* < 0.01] (Table 3).

**Table 3:** Postoperative outcomes and related intraoperative variables.

**Variable**	**Intervention group (*n* = 127)**	**Standard group (*n* = 128)**	***P*-value**
**Postoperative outcome**			
Incidence of delirium within 3 days	10 (7.87)	37 (28.91)	0.000
New cerebral infarctions (symptomatic)^§§^	1 (0.79)	3 (2.34)	0.32
New cerebral infarctions (MRI)^§§^	33 (25.98)	43 (33.59)	0.18
Intracerebral hemorrhage (MRI)	1 (0.79)	2 (1.56)	0.57
Duration of hospital stay after surgery (days)^††^	3.99 ± 1.80	4.26 ± 2.00	0.27
**Intraoperative EEG Suppression**			
Total EEG suppression time (min)^*^^^##^^	0 (0)	0 (1.17)	0.04
Time of EEG suppression in S_1_ (min)^*^^^##^^	0 (0)	0 (0.25)	0.13
Time of EEG suppression in S_2_ (min)^*^^^##^^	0 (0)	0 (0.1)	0.000
Time of EEG suppression in S_3_ (min)^*^^^##^^	0 (0)	0 (0)	0.02

*The values in parentheses are expressed as percentages unless indicated otherwise. Values are expressed as*

* *median (i.q.r.). S_1_ is the stage from induction of anesthesia to clamping the carotid artery, S_2_ from clamping to declamping, and S_3_ from the declamping to the end of surgery.*

§§ *The P value is from the χ^2^ test, except*

†† *The P value is from the independent-samples t test and*

## *The P value is from the independent-samples Mann–Whitney U test*.

### Intraoperative EEG Suppression

Seven patients were excluded from EEG analysis because of corrupted data. The total duration of EEG suppression was significantly shorter in the intervention group than the standard care group [0 (0) *vs*. 0 (1.17), *P* = 0.04] (Table 3). The effect of the intervention on EEG suppression differed according to surgical stage, with significantly shorter durations during S_2_ and S_3_ among the intervention group patients compared to the standard group patients, while there was no significant difference in duration during S_1_.

### Other Outcomes and Safety of the Intervention

There were no significant differences in incidence of new cerebral infarctions according to symptoms and neuroimaging, incidence of intracerebral hemorrhage according to neuroimaging, and hospital stay duration between groups (Table 3). No intraoperative movement or awareness was observed in any patient during surgery and no deaths occurred after surgery.

### Intraoperative Monitoring Values at Different Time-Points

**Figure 2:**
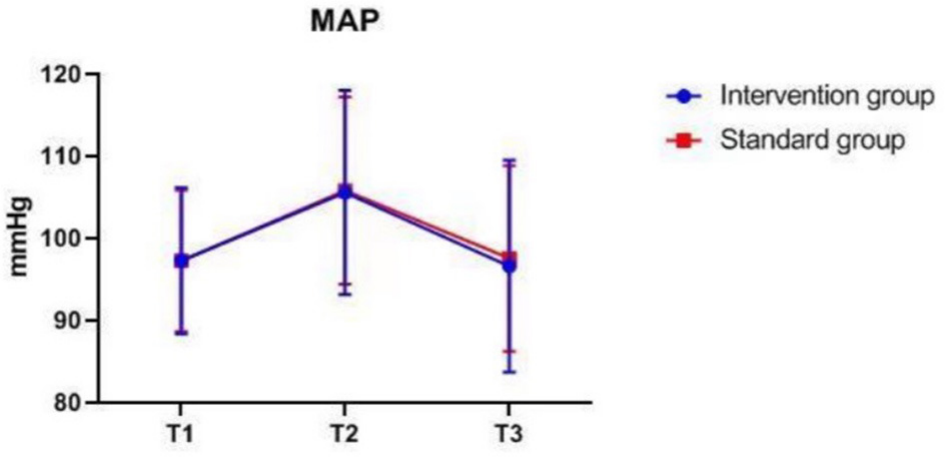
MAP over time. MAP, mean arterial blood pressure. The measurement time interval after general anesthesia but before clamping the carotid artery was recorded as baseline reference T1, the time interval after clamping but before declamping was recorded as T2, and the time interval after the declamping and stabilization of cerebral perfusion but before completion of surgery was recorded as T3.

**Figure 3:**
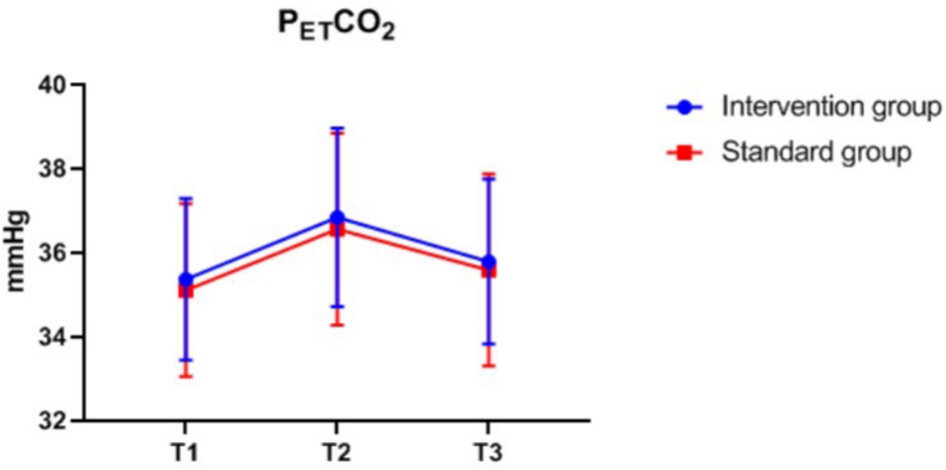
P_ET_CO_2_ over time. P_ET_CO_2_, end-expiratory carbon dioxide partial pressure. The measurement time interval after general anesthesia but before clamping the carotid artery was recorded as baseline reference T1, the time interval after clamping but before declamping was recorded as T2, and the time interval after the declamping and stabilization of cerebral perfusion but before completion of surgery was recorded as T3.

**Figure 4:**
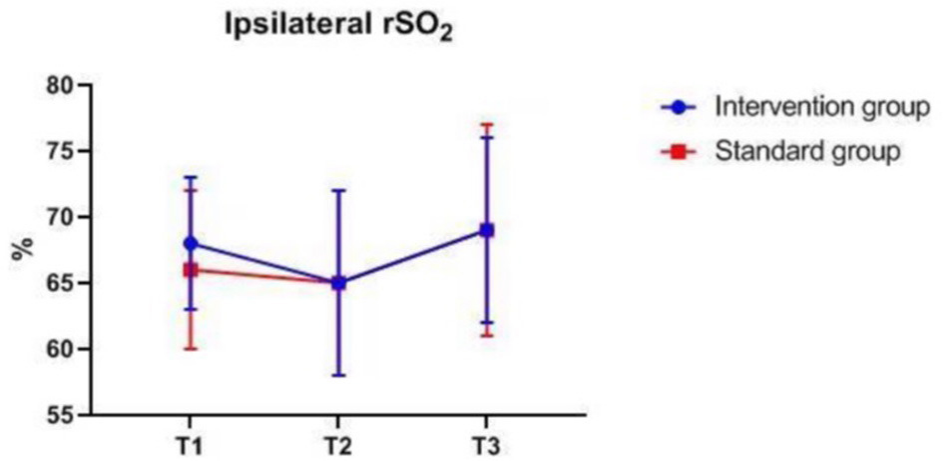
Ipsilateral rSO_2_ over time. rSO_2_, regional cerebral oxygenation. The measurement time interval after general anesthesia but before clamping the carotid artery was recorded as baseline reference T1, the time interval after clamping but before declamping was recorded as T2, and the time interval after the declamping and stabilization of cerebral perfusion but before completion of surgery was recorded as T3.

**Figure 5:**
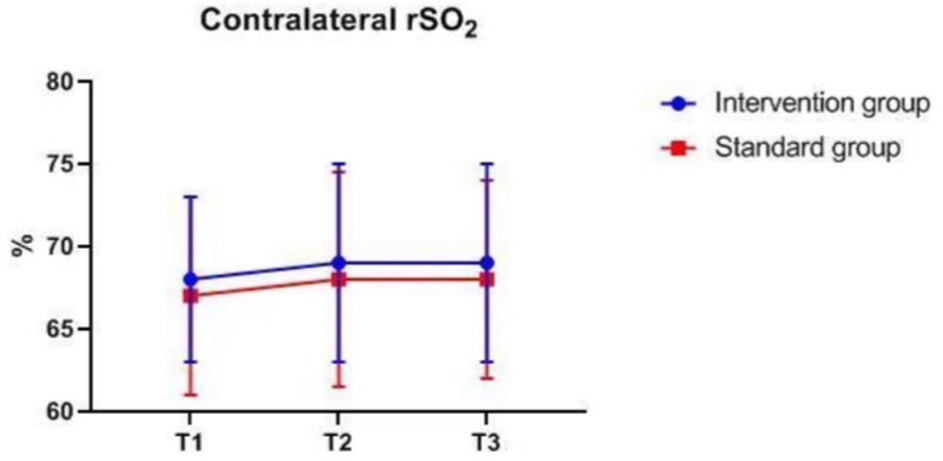
Contralateral rSO_2_ over time. rSO_2_, regional cerebral oxygenation. The measurement time interval after general anesthesia but before clamping the carotid artery was recorded as baseline reference T1, the time interval after clamping but before declamping was recorded as T2, and the time interval after the declamping and stabilization of cerebral perfusion but before completion of surgery was recorded as T3.

**Figure 6:**
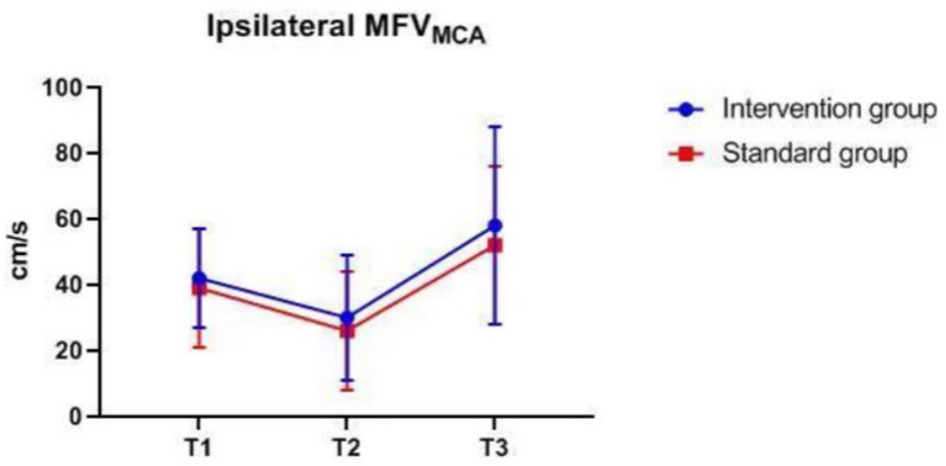
Ipsilateral MFV_MAC_ over time. MFV_MAC_, mean flow velocity of middle cerebral artery. The measurement time interval after general anesthesia but before clamping the carotid artery was recorded as baseline reference T1, the time interval after clamping but before declamping was recorded as T2, and the time interval after the declamping and stabilization of cerebral perfusion but before completion of surgery was recorded as T3. MFV_MAC_, bilateral mean flow velocity of middle cerebral artery.

**Figure 7:**
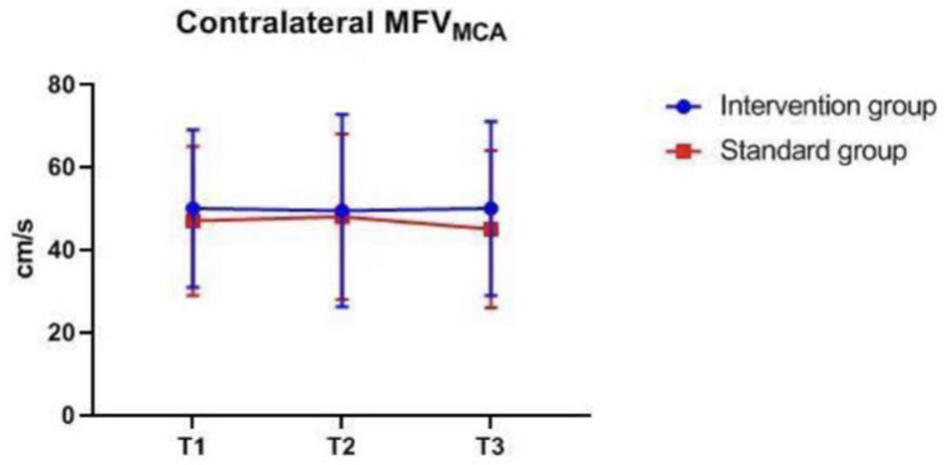
Contralateral MFV_MAC_ over time. MFV_MAC_, mean flow velocity of middle cerebral artery. The measurement time interval after general anesthesia but before clamping the carotid artery was recorded as baseline reference T1, the time interval after clamping but before declamping was recorded as T2, and the time interval after the declamping and stabilization of cerebral perfusion but before completion of surgery was recorded as T3. MFV_MAC_, bilateral mean flow velocity of middle cerebral artery.

**Figure 8:**
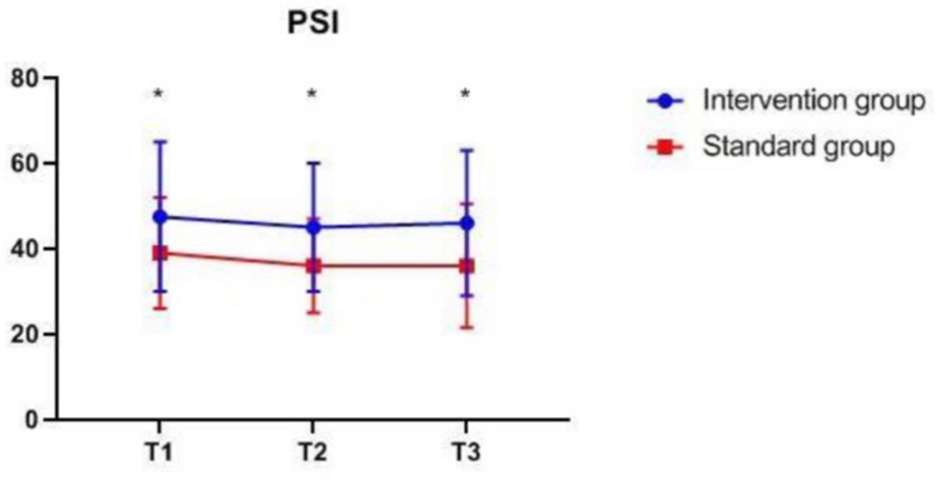
PSI at corresponding time points. PSI, patient state index, *P < 0.05 from standard group (statistically significant). The measurement time interval after general anesthesia but before clamping the carotid artery was recorded as baseline reference T1, the time interval after clamping but before declamping was recorded as T2, and the time interval after the declamping and stabilization of cerebral perfusion but before completion of surgery was recorded as T3.

There were also no significant differences in MAP, P_ET_CO_2_, bilateral MFV_MCA_ and rSO_2_ between groups at corresponding time-points (Figures 2–7), while PSI was higher in the intervention group at all measurement time-points (*P* < 0.05) (Figure 8). There were differences in MAP, P_ET_CO_2_, PSI, ipsilateral MFV_MCA_, and bilateral rSO_2_ at all time-points within the two groups. MAP, P_ET_CO_2_ and contralateral rSO_2_ at T2 were higher than at T1 and T3 (*P* < 0.05); PSI, ipsilateral rSO_2_ and MFV_MCA_ at T2 were lower than at T1 and T3 (*P* < 0.05). While contralateral MFV_MAC_ was not significantly different at all time-points in the two groups (Table 4).

**Table 4:** Intraoperative monitoring values at different time-points.

**Variable**		**Intervention group *n* = 127**	**Standard group *n* = 128**	***P*****-values**	
				**Between groups**	**In the group**
MAP (mmHg)	T1	97.41 ± 9.01	97.36 ± 8.56	0.74	0.000
	T2	105.65 ± 12.36	106.01 ± 11.31		
	T3	96.61 ± 13.09	97.45 ± 11.43		
P_ET_CO_2_ (mmHg)	T1	35.37 ± 1.92	35.16 ± 2.00	0.65	0.000
	T2	36.83 ± 2.09	36.56 ± 2.28		
	T3	35.79 ± 1.96	35.63 ± 2.24		
PSI*	T1	47.50 (17.50)	39.00 (13.00)	0.000	0.02
	T2	45 (15)	36 (11)		
	T3	46.00 (17.00)	36.00 (14.50)		
Ipsilateral rSO${}_{2}^{*}$	T1	68 (5)	66 (6)	0.30	0.000
	T2	65 (7)	65 (7)		
	T3	69 (7)	69 (8)		
Contralateral rSO${}_{2}^{*}$	T1	68 (5)	67 (6)	0.67	0.000
	T2	69 (6)	68 (6.5)		
	T3	69 (6)	68 (6)		
Ipsilateral MFV_MAC_ (cm/s)*	T1	42.00 (15.00)	39.00 (18.00)	0.12	0.000
	T2	30.00 (19.00)	26.00 (18.00)		
	T3	58.00 (30.00)	52.00 (24.00)		
Contralateral MFV_MAC_ (cm/s)*	T1	50.00 (19.00)	47.00 (18.00)	0.11	0.48
	T2	50.0 (22.50)	48.00 (20.00)		
	T3	50.00 (21.00)	45.00 (19.00)		

Values are expressed as mean ± s.d. or *median (i.q.r.). The measurement time interval after general anesthesia but before clamping the carotid artery was recorded as baseline reference T1, the time interval after clamping but before declamping was recorded as T2, and the time interval after the declamping and stabilization of cerebral perfusion but before completion of surgery was recorded as T3. MAP, mean arterial blood pressure; PSI, patient state index; P_ET_CO_2_, end-expiratory carbon dioxide partial pressure; rSO_2_, bilateral regional cerebral oxygenation; MFV_MAC_, bilateral mean flow velocity of middle cerebral artery.

*The P-value is from the RT-ANOVA. The P-value is from the RT-ANOVA*.

## Discussion

We demonstrated that in patients undergoing CEA, processed EEG-guided anesthesia management with PSI combined with DSA reduces the incidence of POD compared to using PSI alone. Based on previous studies on POD (11, 14, 17–19), we suggest that not only the index from a processed EEG but also an index with additional values (in this case, DSA) to guide anesthesia management protocol may improve outcomes among CEA patients.

The SEDLine monitor provides computed quantitative EEG indices based on retrospective analysis of a diagnostic EEG database of sedated patients. Since regulatory approval in 2002, these indices have been shown to independently predict deep sedation as assessed by other clinical metrics such as the Ramsay Sedation Score and Modified Observer's Assessment of Alertness/Sedation Scale (52, 63–65). Our findings of reduced POD incidence using processed EEG-guided anesthesia management are in accord with previous studies on other surgical populations (27–29, 66, 67). In contrast, the Electroencephalography Guidance of Anesthesia (ENGAGES) trial of older adults undergoing cardiac or non-cardiac surgery found no reduction in delirium incidence using this EEG-based intervention (68). Possible explanations for this discrepancy include differences in baseline conditions between cohorts and the unique effects of cardiopulmonary bypass on the cerebrum. A comprehensive description of predisposing factors is critical for investigations on POD, especially those including patients with multiple etiologies. Leung and colleagues recently reported the results of their clinical trial in older adults undergoing non-cardiac surgery, and reported similar rates of postoperative delirium between patients randomized to receive EEG-guided anesthetic management vs. those without the monitor (66). Unlike our study, most of the patients included in this study were undergoing spinal surgery, and the surgical process had little effect on the cerebral circulation. In contrast to the two trials, our patient groups were well-matched demographically and clinically at baseline, and received similar surgical procedures with comparable impacts on cerebral circulation, permitting similar anesthesia management (except for the EEG-based intervention). Another possible explanation for this discrepancy is that anesthesia requirements may be highly patient-specific due to individual sensitivity to anesthetic depth (69). EEG suppression is also a function of cerebral perfusion. The patients recruited in our study all had severe carotid artery stenosis and exhibited baseline characteristics predictive of greater sensitivity to anesthesia depth and POD compared to the two cohort. Thus, patient vulnerability is an important consideration when identifying patients most suitable for processed EEG monitoring. A slight change in anesthetic management may play a major role in modulating postoperative delirium risk in patients with cerebral ischemia (or patients at a risk of cerebral ischemia).

In this study, both groups of patients used the index from a processed EEG, and the intervention group combined the index with additional values (in this case the DSA) to guide anesthesia. It was observed that using the PSI alone did not result in as good outcomes and BS avoidance, possibly due to underestimation of EEG suppression using the monitoring (56). Patients may have benefited from BSR and PSI monitoring, visual tracking of the EEG, and the individualized real-time feedback of an anesthetic cerebral state provided by the EEG spectrogram or raw waveform patterns (70–72), thereby achieving superior anesthesia depth monitoring and reducing POD risk. Anesthesiologists may be able to manage patients' anesthetic cerebral state better by monitoring multiple EEG parameters. The occurrence of EEG burst suppression during maintenance may be predictive of POD (73). Lower EEG suppression duration may have directly contributed to the reduced POD incidence in the intervention group; hence, vulnerable patients may have derived a greater benefit. The PSI of the intervention group is higher than that of the standard care group, however, if only a single PSI index is observed and maintained at a high level, EEG suppression may not be minimized, which may not be enough to significantly reduce the risk of POD.

The EEG suppression is also a function of cerebral perfusion, in other words, the lower the perfusion, the more suppressed the EEG. Our patients had severe carotid artery stenosis and relatively insufficient cerebral perfusion. The severe fluctuations of cerebral blood flow (CBF) during CEA may have further aggravated hypoperfusion or led to cerebral hyperperfusion. In our study, the combined monitoring of TCD and NIRS may minimize the influence of the patient's hemodynamics on cerebral perfusion. However, the largest changes in CBF occurred during S_2_ and S_3_, and the duration of EEG suppression was significantly lower during these stages in the intervention group. In the intervention group, while adjusting the anesthesia dose to reduce EEG burst suppression, we also paid close attention to the effect on CBF. When adjusting the anesthetic dose, burst suppression could not be minimized if CBF changes were unfavorable. Therefore, even if the TCD and NIRS monitors did not show hypoperfusion or hyperperfusion, we still slightly regulated blood pressure according to the surgical stage. Although we observed no difference in MAP at each time point between groups, small adjustments may have slightly improved CBF, reduce EEG suppression, and thereby reduce POD.

Anesthetic dosage can be reduced using processed EEG (27, 74), and previous studies have shown potentially neurotoxic effects of general anesthetics, including propofol (75, 76). Therefore, it is conceivable that POD risk was reduced by lower anesthetic levels. However, the present results do not support this premise, at least for the anesthetic doses used and for this specific population. All patients had severe CS and were prone to vascular events which had been identified as one of the postoperative risk factors for the development of delirium. In addition to adjusting the anesthetic dose during the perioperative period, we also adjusted blood pressure according to the operation stage to avoid potential cerebral perfusion problems that may lead to burst suppression. Moreover, we conducted multimodal monitoring on all patients, which helped us obtain more comprehensive information to identify the extent of individual cerebral perfusion. We adjusted the physiological functions of patients according to anesthetics/techniques and surgical procedures, and strictly controlled arterial blood pressure (77) to avoid insufficient or excessive cerebral perfusion risk related to POD. All the monitoring we used is non-invasive and has no significant risk of injury to patient. Therefore, we conclude that patients with carotid artery stenosis can benefit from multimodal monitoring during surgery to reduce the onset of EEG suppression.

The difference in MAP, P_ET_CO_2_, ipsilateral MFV_MCA_, and bilateral rSO_2_ at all time-points is due to differences in blood pressure management measures taken at different surgical stages to ensure that the cerebral perfusion is within desired limits. Despite the difference in contralateral rSO_2_, there was no difference in MFV_MCA_, likely because TCD monitoring reflects MFV_MCA_, while NIRS monitors the oxygenation of the frontal cortex mainly supplied by the anterior communicating artery (ACA). Since the contralateral MFV_MCA_ is not affected by the surgery, there is no difference at all time-points. The NIRS monitoring value may have been influenced by oxygen metabolism of extracranial origin as well as by changes in blood pressure and arterial oxygen saturation. The differences in PSI within group at corresponding time-points are likely because the largest changes in CBF during clamping and declamping of the carotid artery may also affect EEG, leading to changes in PSI.

### Potential Limitations

The study has several limitations. First, as a single-center trial, the generalizability of the results may be limited. Second, postoperative delirium has no objective biomarker and so may be difficult to diagnose in certain cases (78). Third, POD was assessed only over 3 days, so transient and later incidences could have been missed. However, the preponderance of evidence suggests that most cases occur within the first 3 days after anesthesia and surgery (14, 79). Fourth, the anesthesiologists were not blinded to group assignment; therefore, when caring for patients in the intervention group, they might have been more cautious in the management of the patient's overall hemodynamic parameters. However, the following measures were adopted to avoid bias. The combined monitoring of TCD and NIRS may minimize the influence of the patient's hemodynamics on cerebral perfusion. There was no difference in MAP, bilateral MFV_MCA_ and rSO_2_ at different time-points between the two groups. The anesthetists did not participate in postoperative follow-up, and the investigators responsible for postoperative follow-up and delirium assessments were blinded to group assignment and did not participate in anesthesia or perioperative care. The anesthetists and investigators also did not communicate patient information. Fifth, these findings may not apply to patients receiving inhalational anesthetics.

## Summary

This randomized clinical trial suggests that electroencephalography-guided anesthesia management with both a quantitative EEG index and DSA can reduce postoperative delirium incidence in patients undergoing CEA, especially those vulnerable to disruption of cerebral perfusion and EEG suppression. A slight change in anesthetic management may play a significant role in modulating postoperative delirium risk in patients with ischemic cerebral disease (or patients at risk of cerebral ischemia). In turn, reducing POD incidence may improve outcome.

## Data Availability Statement

The original contributions presented in the study are included in the article/supplementary material, further inquiries can be directed to the corresponding author/s.

## Ethics Statement

The studies involving human participants were reviewed and approved by the Ethics Committee of Xuanwu Hospital of Capital Medical University (LYS[2018]053). The patients/participants provided their written informed consent to participate in this study.

## Author Contributions

NX was responsible for the implementation of the project, patient recruitment, patient interviews, data collection, data entry, statistical analyses, and article writing. L-XL, D-XY, JW, Y-HM, TT, and X-LS performed patient recruitment, patient interviews, and data collection. L-QJ and YH reviewed and edited the final manuscript. T-LW managed the project and provided funding. All authors contributed to manuscript revision, read, and approved the submitted version.

## Conflict of Interest

The authors declare that the research was conducted in the absence of any commercial or financial relationships that could be construed as a potential conflict of interest.

## Abbreviations

CEA: carotid endarterectomy
EEG: electroencephalogram
TCD: transcranial Doppler ultrasound
NIRS: near-infrared spectroscopy
CS: carotid stenosis
POD: postoperative delirium
CAM: Confusion Assessment Method
MMSE: Mini-Mental State Examination
MoCA: Montreal Cognitive Assessment
SDS: Self-rating Depression Scale
SAS: Self-rating Anxiety Scale
ASA: American Society of Anesthesiologists
rSO_2_: regional cerebral oxygenation
MFV_MCA_: mean flow velocity of the middle cerebral artery
BSR: burst suppression ratio
DSA: density spectral array
PSI: patient state index
MAP: mean arterial blood pressure
P_ET_CO_2_: end-expiratory carbon dioxide partial pressure
SD: standard deviation
IQR: interquartile range
OR: odds ratio
ACA: anterior communicating artery
CBF: cerebral blood flow
CAS: carotid artery stenting
EMG: electromyogram.
